# Timing of Ethylene Inhibition Affects Internal Browning and Quality of ‘Gala’ Apples in Long-Term Low Oxygen Storage

**DOI:** 10.3389/fpls.2022.914441

**Published:** 2022-05-30

**Authors:** Jennifer R. DeEll, Geoffrey B. Lum, Younes Mostofi, Sky K. Lesage

**Affiliations:** Ontario Ministry of Agriculture, Food and Rural Affairs, Simcoe, ON, Canada

**Keywords:** *Malus×domestica*, 1-MCP, storage disorder, dynamic CA, SafePod

## Abstract

The objective of this study was to evaluate the timing of ethylene inhibition with preharvest and postharvest 1-methylcyclopropene (1-MCP) treatments on internal browning and quality of ‘Gala’ apples in long-term low O_2_ storage. ‘Gala’ apples were obtained from the same commercial orchard during their harvesting period for 2 years of study. Preharvest 1-MCP orchard spray (3.8% a.i) was applied at the label rate of 60 g 1-MCP per acre in the first year. Postharvest 1-MCP (1 μl L^−1^) treatments were made for 24 h at 0.5°C either at harvest time (1 day after harvest) or after storage in controlled atmosphere (CA) in both years. Apples were stored in 1.5 kPa O_2_ + 0.5 kPa CO_2_ or 0.6 kPa O_2_ + <0.5 kPa CO_2_ for 9 months in the first year and 1.5, 1.0, or 0.5 kPa O_2_ + 0.5 kPa CO_2_ for 8 months in the second year. Storage regimes with O_2_ concentrations less than 1 kPa were based on fruit respiration using SafePod™ technology. After removal from storage, all apples were then evaluated for internal browning and other quality attributes after 1, 7, and 14 days at room temperature (RT, 21–22°C). Internal browning developed in ‘Gala’ apples during both years of study, with up to 16% incidence across treatments in the first year and up to 84% in the second year. Apples stored in 0.5–0.6 kPa O_2_ had significantly less internal browning during both years of study, compared to apples stored in higher O_2_. The effect of 1-MCP on internal browning was negligible in 0.5–0.6 kPa O_2_ storage. ‘Gala’ stored in 1.5 kPa O_2_ and treated with postharvest 1-MCP after storage had significantly less internal browning with preharvest 1-MCP than those without preharvest treatment. Apples treated with postharvest 1-MCP at harvest time, instead of after storage, did not exhibit this same effect. Preharvest 1-MCP-treated fruit maintained greater firmness retention than those without preharvest 1-MCP, and this effect was further enhanced when 1-MCP was applied after storage. Postharvest 1-MCP had no effect on firmness retention in fruit without preharvest 1-MCP, but lower O_2_ maintained greater firmness in those apples. Preharvest 1-MCP had no significant effect on internal ethylene concentration, whereas it was reduced by postharvest 1-MCP at harvest time in the first year of study, regardless of storage regimes. However, internal ethylene was only affected by storage regime in the second year, with lower concentration in fruit from 0.5 kPa O_2_ than in those from higher O_2_. Greasiness developed only in the second year and postharvest 1-MCP consistently reduced it, regardless of treatment timing and storage regime. There was no greasiness in apples treated with postharvest 1-MCP at harvest and then held in 0.5 kPa O_2_ for 8 months plus 14 days at room temperature. Soluble solids concentration and malic acid content were slightly higher in ‘Gala’ apples with preharvest 1-MCP compared to those without, whereas there was little and inconsistent effect of postharvest 1-MCP on these attributes. Overall, storage regimes with less than 1 kPa O_2_ provided the least amount of internal browning and best quality attributes. Ethylene inhibition provided further benefits, but this was dependent on the timing of 1-MCP treatment.

## Introduction

1-Methylcylcopropene (1-MCP) is a competitive inhibitor of ethylene action. Postharvest treatment of apples with 1-MCP is well documented to improve retention of quality characteristics, including reduced ethylene production and respiration, as well as improved firmness and acidity retention (DeEll et al., 2005, 2007; Watkins, 2007). The efficacy of 1-MCP on apples can be affected by various factors, including cultivar (Watkins et al., 2000; Bai et al., 2005), fruit maturity at harvest (Toivonen and Lu, 2005; DeEll et al., 2008), duration and temperature of exposure (DeEll et al., 2002), timing of application (DeEll et al., 2008, 2012b; Watkins and Nock, 2012), concentration and number of applications (Nock and Watkins, 2013; DeEll et al., 2016b), and delays in application after harvest (Watkins and Nock, 2005; DeEll et al., 2008). Studies have demonstrated postharvest 1-MCP treatment can alleviate certain physiological disorders in apples after several months of storage (Watkins et al., 2000; DeEll et al., 2002, 2008). However, 1-MCP can also exacerbate the development of disorders, such as internal browning (Jung and Watkins, 2011) and CO_2_ injury (DeEll et al., 2003; Fawbush et al., 2008) in apples.

Application of 1-MCP prior to harvest can also have an impact on apple quality retention and disorders during storage. ‘Fuji’ apples treated with only preharvest 1-MCP maintained higher firmness and titratable acidity levels, and lower incidence of stem-end browning compared to untreated fruit after cold storage at 0.5°C for up to 36 weeks (Lee et al., 2019). Moreover, preharvest 1-MCP treatment can reduce soft scald development in ‘Honeycrisp’ after 5–6 months of air storage (DeEll and Ehsani-Moghaddam, 2010). Argenta et al. (2018) found preharvest 1-MCP slowed softening and reduced severity of disorders associated with harvest delay in ‘Gala’ apples, but this was dependent on orchard, ‘Gala’ strain, and storage regime. Studies evaluating the influence of preharvest 1-MCP treatment alone or in combination with postharvest 1-MCP on storage disorders in apples under low O_2_ conditions are limited.

Internal browning is characterized by diffuse browning of apple flesh tissue and is typically not visible from the external surface (Meheriuk et al., 1994; DeEll et al., 2007; Watkins and Liu, 2010). The first sign of internal browning in ‘Gala’ usually involves radial flesh browning near the stem-end (shoulder), which can progress toward the calyx end of the fruit (Lee et al., 2013, 2016). Apples with advanced maturity at harvest or prolonged period in storage and increased duration at room temperature after storage tend to have higher incidence of internal browning and susceptibility to storage-related disorders (Watkins et al., 2000; DeEll et al., 2016c). Many factors can influence the onset of internal browning in apples, including the orchard system, orchard management practices, growing seasons, fruit maturity at harvest, postharvest treatments, storage conditions and storage duration (Ehsani-Moghaddam and DeEll, 2009; Jung and Watkins, 2011; DeEll and Ehsani-Moghaddam, 2012b; DeEll et al., 2016b,c). Nonetheless, the exact biological processes and mechanisms associated with the development of internal browning disorder in apple during and after storage remains to be elucidated.

Advancement of controlled atmosphere technologies has allowed apples to be stored for historically longer periods and readily available year-round for consumers. Apples are commonly stored in low O_2_ (1–3 kPa) and elevated CO_2_ (1–3 kPa) partial pressures under low temperature to maintain fruit quality characteristics and storage-life, and limit metabolic processes associated with fruit ripening (Brackmann et al., 1993; Mattheis et al., 2005; Lee et al., 2012). There has been a renewed interest in low O_2_ concentrations for apple storage and regimes with less than 2 kPa O_2_ have shown many advantages in maintaining fruit quality, including reduced ethylene production and respiration, improved fruit firmness retention, sugars and acidity levels, and delayed fruit senescence (Köpcke, 2015; Rebeaud and Gasser, 2015; Thewes et al., 2015, 2021; Both et al., 2016). Low O_2_ storage can also alleviate symptoms of superficial scald in certain susceptible apple cultivars (Poirier et al., 2020). However, the introduction of low O_2_ levels less than 2 kPa can also exacerbate the development of low O_2_-related stress and internal browning injuries in apples (Wright et al., 2015).

Dynamic controlled atmosphere (DCA) storage in ultra-low O_2_ (ULO) conditions (<1 kPa O_2_) is an emerging strategy for extending storage-life and reducing the development of internal browning in apples. DCA storage involves the reduction of O_2_ partial pressures to the lowest possible tolerance level without inducing excess anaerobic metabolism, which will affect fruit quality and increase the presence of fermentation products and off-flavors (Thewes et al., 2015; Bessemans et al., 2016). With the application of DCA storage, monitoring fruit stress can be conducted in real-time through the measurements of ethanol production or respiration rate.

One approach for evaluating low O_2_-related stress in fruit is by the fruit respiratory quotient (RQ); the ratio of CO_2_ production to O_2_ consumption (Gran and Beaudry, 1993; Yearsley et al., 1996). The integration of RQ measurements within a storage system can allow autonomous adjustments of atmospheric composition when low O_2_-related stress is detected during the storage period. Commercial RQ-based DCA respiration chambers (SafePod™) were first shown to reduce internal browning in ‘Empire’ apples treated postharvest with 1-MCP and held in less than 1 kPa for 8 months (DeEll and Lum, 2017). More recently, protocols were developed to determine low oxygen limits and monitor the response of ‘Braeburn’ and ‘Gala’ apples to low O_2_ in large-scale storages, using the same SafePod™ technology (Rees et al., 2021). Other reports further demonstrated the application of DCA-based strategies for maintaining apple quality and limiting storage-related disorders. ‘Braeburn’ apples treated postharvest with 1-MCP and held in DCA storage had higher maintenance of fruit firmness compared to similar fruit held in CA storage; however, there was no difference on incidence of flesh breakdown across the different storage regimes (Schmidt et al., 2020). ‘Royal Gala’ apples stored in 0.7, 0.4, and 0.15 kPa O_2_ had lower ethylene production than fruit stored in 1.2 kPa O_2_, regardless of CO_2_ levels (Thewes et al., 2021). Moreover, ‘Royal Gala’ apples stored in DCA-chlorophyll fluorescence (CF) with a range of 0.8–0.4 kPa O_2_ had comparable ethylene production as fruit stored in static CA with 0.4 kPa O_2_ or 0.15 kPa O_2_ after removal from storage (Thewes et al., 2021). RQ response has been shown to be consistent with increases in CF yield during DCA storage of ‘Braeburn’ and ‘Gala’ apples (Rees et al., 2021).

Some benefits of low O_2_ storage for ‘Gala’ apples have been documented in the scientific literature (Both et al., 2014, 2016, 2017; Thewes et al., 2015, 2021). However, the response of ‘Gala’ apples to the timing of ethylene inhibition with preharvest and postharvest 1-MCP treatments in combination with low O_2_ storage is not well understood. The objective of this study was to evaluate the timing of ethylene inhibition with preharvest and postharvest 1-MCP treatments on internal browning and quality of ‘Gala’ apples in long-term low O_2_ storage. Regimes using <1 kPa O_2_ were based on RQ-DCA (SafePod™ technology). Postharvest 1-MCP treatments were either at harvest time or after storage.

## Materials and Methods

### Plant Material and Treatments

Trees in a mature ‘Gala’ (Imperial) apple orchard on M.9 rootstock were selected within a commercial orchard in Norfolk County, Ontario, Canada. In 2019 (Year 1), six individual rows of 30 trees were randomly flagged for the experiment, with four rows (not flagged) in between each flagged row for a buffer to any spray drift. Three of these flagged rows were sprayed with 1-MCP (3.8% a.i.; Harvista™; AgroFresh Inc., Philadelphia, Pennsylvania, United States) at the label rate of 60 g 1-MCP per acre using a commercial turbo mist sprayer. The remaining three flagged rows were not sprayed. Seven days after spraying, six boxes of fruit were harvested from each row and each box contained ~80 fruit from several trees and from various locations within the trees of that row. This made for a total of 18 boxes (6 boxes × 3 orchard rows) with 1-MCP orchard spray and 18 boxes without the spray. All apples were transported within 1 h of harvest to the nearby apple storage research facility and cooled overnight at 0.5°C. The next day two boxes from each row with and without 1-MCP orchard spray were treated with 1-MCP (1 μl L^−1^) for 24 h, using SmartFresh™ tablets (AgroFresh Inc.) within air-tight aluminum CA storage chambers (Storage Control Systems Inc., Sparta, Michigan, United States) at 0.5°C. Thereafter, one box with the postharvest 1-MCP treatment along with two boxes without postharvest 1-MCP from each orchard row (with and without 1-MCP orchard spray), were placed into CA storage of 1.5 kPa O_2_ + 1 kPa CO_2_ and similarly into 0.6 kPa O_2_ + <0.5 kPa CO_2_ at 0.5°C for 9 months. After removal from both storage regimes, one box from each row with and without 1-MCP orchard spray that had not been previously treated with postharvest 1-MCP was then treated with 1-MCP (1 μl L^−1^, SmartFresh™ tablets) for 24 h at 0.5°C. This resulted in three boxes (1 box × 3 orchard rows) per treatment combination—with and without 1-MCP orchard spray, and treated with postharvest 1-MCP at harvest time, after storage, or not at all—for a total of 18 boxes per storage regime. In 2020 (Year 2), 27 boxes were harvested from the same orchard but there was no preharvest 1-MCP orchard spray. As in the previous year, three boxes were treated with postharvest 1-MCP at harvest time, after storage, or not at all. There were three low O_2_ regimes, which consisted of 1.5, 1.0, or 0.5 kPa O_2_ + 0.5 kPa CO_2_ at 0.5°C for 8 months.

The CA storage system consisted of aluminum storage chambers (0.9 m^3^ volume) fitted with a circulating fan system (Storage Control Systems Inc.). Atmospheres were checked hourly and maintained within 0.1 kPa of target values using an ICA 61/CGS 610 CA Control System (International Controlled Atmosphere Ltd., Kent, United Kingdom), which was modified with flow controllers for the chambers (Storage Control Systems Inc.). Storage regimes with O_2_ concentrations < 1 kPa were based on fruit respiration using SafePod™ technology (Rees et al., 2021) and connected to the same ICA 61/CGS 610 CA Control System. Fruit respiration of ‘Gala’ with and without preharvest 1-MCP orchard spray was also measured for 6 days immediately after harvest at room temperature using this system in the first year of study.

### Fruit Quality and Disorder Evaluations

Initial fruit maturity at the time of harvest was evaluated using 10-apple samples from each replicate (orchard row). Internal ethylene concentration was determined by withdrawing a 3-ml gas sample from the core of each fruit using a syringe and injecting the gas sample into a Agilent 7820A gas chromatograph (Agilent Technologies Canada Inc., Mississauga, Ontario, Canada) equipped with a 0.25 ml sample loop, flame ionization detector, and 25 m × 0.53 mm CarboBOND capillary column (Agilent Technologies Canada Inc.). The injector, column, and detector temperatures were 150, 80, and 250°C, respectively. High-purity helium was used as the carrier gas at a flow rate of 0.46 ml s^−1^ with a typical run time of 1.5 min.

Fruit firmness was determined on opposite sides of each apple after peeling, using an electronic texture analyzer fitted with an 11-mm tip (GÜSS, South Africa). Titratable acidity (expressed as mg equivalents of malic acid per 100 ml of juice) was determined by titrating a 2-ml juice sample (extracted from composite samples of fragments from all apples used for firmness testing) with 0.1 N NaOH to an end point of pH 8.1 (as indicated by phenolphthalein). Soluble solids concentration was determined on a similar juice sample using a digital refractometer (PR-32; Atago Co. Ltd., Japan). Starch index values were determined using the Generic Starch-Iodine Index Chart for Apples (Blanpied and Silsby, 1992). Apples were cut in half at the equator, dipped in potassium-iodine solution and rated on a scale of 1–8, where 1 = 100% starch staining and 8 = no starch.

After removal from CA storage, fruit were held at room temperature (RT, 21–22°C) and then evaluated for fruit quality and storage disorders after 1, 7, and 14 days. Ten fruit from each of three boxes for each treatment combination were measured for internal ethylene concentration, firmness, soluble solids concentration, and malic acid content. The incidence of storage disorders, namely internal or stem-end browning, were determined using all apples in each box (~80 fruit per box × 3 boxes per treatment combination). Incidence was calculated as a percentage of fruit displaying the disorder regardless of severity. Greasiness was evaluated as reported in DeEll et al. (2016a), using 1 = none to slight greasiness; 2 = moderate greasiness (noticeable by feel); and 3 = severe greasiness (slippery, difficult to hold).

### Statistical Analyses

Data from each year were analyzed using Proc GLM and Proc GLIMMIX of the SAS program (version 9.2; SAS Institute Inc., Cary, NC), incorporating a split-plot experimental design. All data were subjected to testing of normality and assumptions for ANOVA, and transformed for analysis when appropriate. Mean separations were examined using Duncan’s multiple range test and only differences significant at *p* ≤ 0.05 are discussed.

## Results

**Figure 1:**
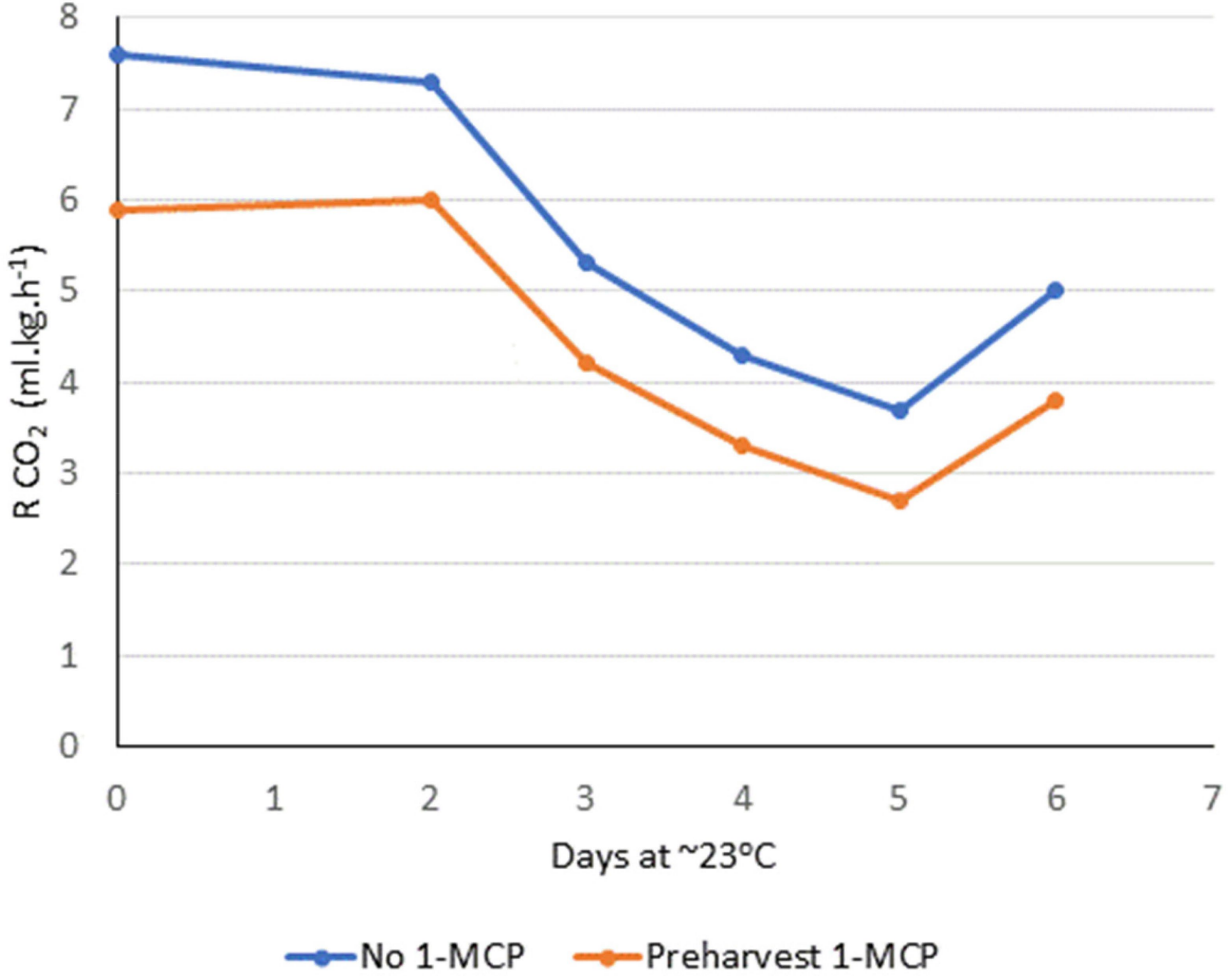
Respiration rate of ‘Gala’ apples without or with preharvest 1-MCP held for 6 days at room temperature (~23°C) immediately after harvest, as measured by CO_2_ production using SafePod™ technology.

‘Gala’ apples were obtained during the commercial harvest period and fruit maturity is presented in Table 1. Treatment with preharvest 1-MCP resulted in firmer fruit and lower starch index values at harvest. There were no significant differences in internal ethylene concentration, soluble solids, and malic acid content at harvest between apples treated with or without preharvest 1-MCP. All apples were considered marketable at harvest with no visible symptoms of disorders. ‘Gala’ apples with preharvest 1-MCP treatment appeared to have reduced respiration immediately after harvest (Figure 1).

**Table 1:** Maturity indices of ‘Gala’ apples at harvest time.

	Internal ethylene concentration (μl L^−1^)	Firmness (*N*)	Soluble solids concentration (%)	Malic acid^1^ (mg)	Starch index^2^ (1–8)
**2019**					
No preharvest 1-MCP	1.9	82.8	11.4	375	5.3
+ Preharvest 1-MCP	1.6	89.1	11.2	339	3.8
*Significance* *^3^*	*NS*	****	*NS*	*NS*	***
**2020**					
No preharvest 1-MCP	0.8	78.8	12.4	331	6.4

1 mg per 100 ml of juice.

2 Based on the Generic Starch-Iodine Index Chart for Apples (Blanpied and Silsby, 1992).

3 NS, ***, **** = not significant or significant at *p* < 0.001 or *p* < 0.0001, respectively.

**Figure 2:**
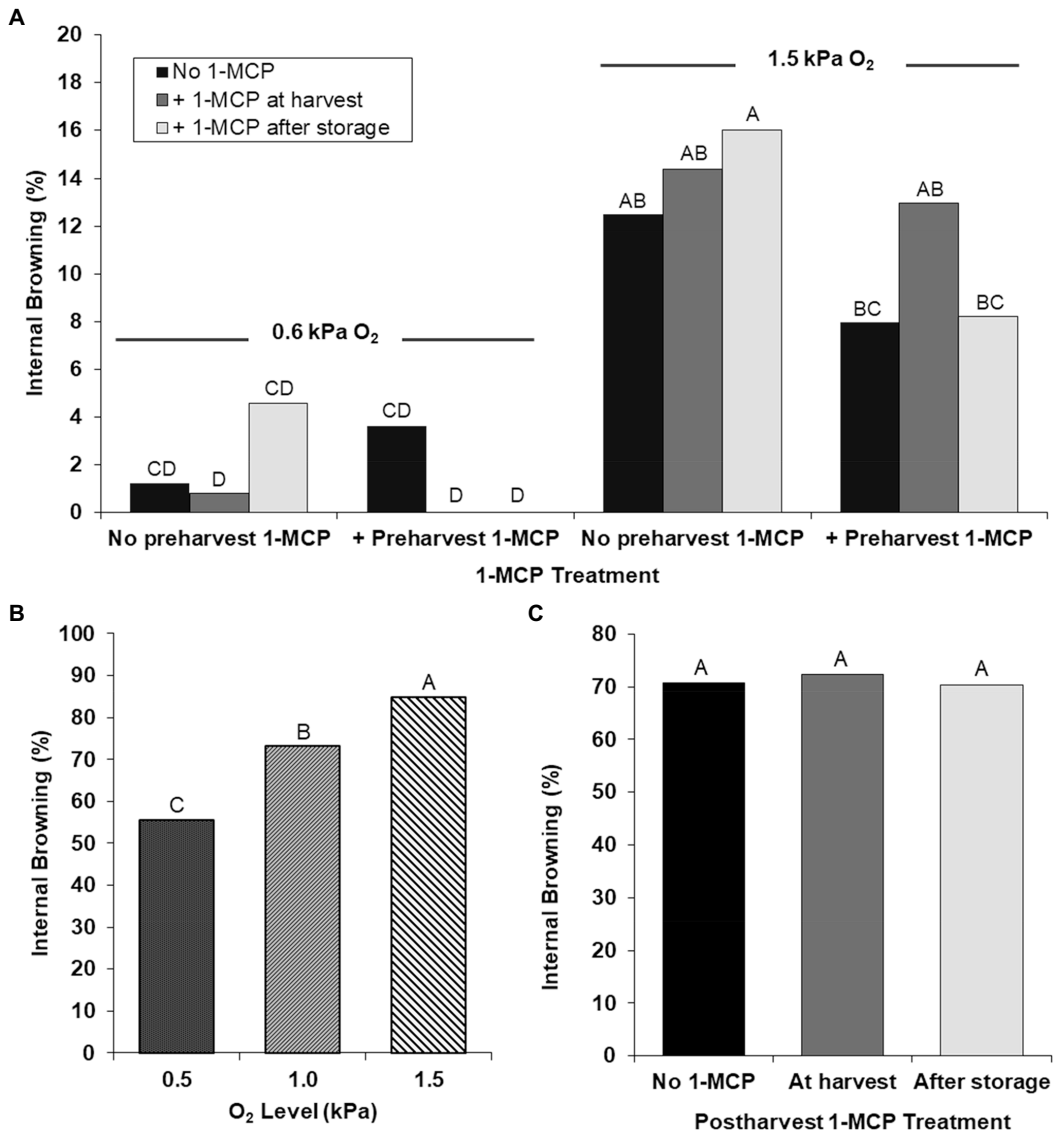
Internal browning of ‘Gala’ apples: **(A)** treated without or with preharvest 1-MCP at 7 days before harvest in combination without or with postharvest 1-MCP (1 μl L^−1^) for 24 h at harvest or after storage with 1.5 or 0.6 kPa O_2_ + <0.5 kPa CO_2_ at 0.5°C for 9 months plus 14 days at room temperature (RT, 21–22°C) in Year 1; **(B)** overall effect of low O_2_ when stored in 1.5, 1.0, or 0.5 kPa O_2_ + <0.5 kPa CO_2_ for at 0.5°C for 8 months plus 14 days at RT in Year 2; and **(C)** overall effect of postharvest 1-MCP when treated without or with postharvest 1-MCP (1 μl L^-1^) for 24 h at harvest or after storage with 1.5, 1.0, or 0.5 kPa O_2_ + <0.5 kPa CO_2_ at 0.5°C for 8 months plus 14 days at RT in Year 2. Values within a graph with the same letter are not significantly different at *p* < 0.05.

Internal browning developed during both years of study, with up to 16% incidence across treatments in the first year and up to 84% in the second year. Apples stored in 0.5–0.6 kPa O_2_ had significantly less internal browning during both years of study, compared to apples stored in higher O_2_ (Figures 2A,B). The effects of preharvest and postharvest 1-MCP treatments on internal browning were negligible in 0.5–0.6 kPa O_2_ storage. ‘Gala’ stored in 1.5 kPa O_2_ and treated with both preharvest 1-MCP and postharvest 1-MCP after storage had less internal browning than similar apples without preharvest 1-MCP (Figure 2A). Apples treated with postharvest 1-MCP at harvest time, instead of after storage, did not exhibit this same effect. There was notably more internal browning in the second year of study and apples stored in lower O_2_ regimes had lower incidence (Figure 2B). Postharvest 1-MCP treatments at harvest or after storage had no significant effects on internal browning in ‘Gala’ apples (Figure 2C), which was similar to the first year of study when no preharvest 1-MCP was applied (Figure 2A). Few apples had only stem-end browning, so these were included in the internal browning incidence.

During the first year of study, apples with preharvest 1-MCP remained firmer than those not treated after 9 months of storage, with overall firmness of 81.5 and 77.7 N, respectively. Furthermore, this effect was present in apples from both storage regimes (Table 2). In contrast, there was no overall significant effect of postharvest 1-MCP treatment on firmness, with average firmness ranging from 79.2 to 79.7 N. There was also no significant interaction of postharvest 1-MCP and storage regime on fruit firmness after 9 months of storage (data not presented).

**Table 2:** Firmness of ‘Gala’ apples treated without or with preharvest 1-methylcyclopropene (1-MCP) orchard spray at 7 days before harvest and then stored in 1.5 or 0.6 kPa O_2_ + <0.5 kPa CO_2_ at 0.5°C for 9 months in Year 1.

	Firmness (*N*)
**1.5 kPa O_2_**	
No preharvest 1-MCP	77.0^C^
+ Preharvest 1-MCP	81.9^A^
**0.6 kPa O_2_**	
No preharvest 1-MCP	78.3^B^
+ Preharvest 1-MCP	81.5^A^
*Significance* *^1^*	****

Values are averages from after storage plus 1, 7, and 14 days at room temperature (21–22°C).

1 **** = significant at *p* < 0.0001.Values within a column with the same letter are not significantly different at *P* < 0.05.

Apples with no preharvest 1-MCP held in 0.6 kPa O_2_ were firmer than those held in 1.5 kPa O_2_ after 9 months of storage (Table 2). In contrast, this effect was not present when fruit were treated with preharvest 1-MCP and held in either storage regime. Fruit with postharvest 1-MCP after storage in combination with preharvest 1-MCP were firmer than those not treated with postharvest 1-MCP (Table 3). Without preharvest 1-MCP, there were no significant differences in retained firmness of apples treated without or with postharvest 1-MCP, regardless of timing at harvest or after storage.

**Table 3:** Firmness of ‘Gala’ apples treated without or with preharvest 1-MCP orchard spray at 7 days before harvest in combination without or with postharvest 1-MCP (1 μl L^-1^) for 24 h at harvest or after storage in 1.5 or 0.6 kPa O_2_ + <0.5 kPa CO_2_ at 0.5°C for 9 months in Year 1.

	Firmness (*N*)
**No preharvest 1-MCP**	
No 1-MCP	77.7^C^
+ 1-MCP at harvest	77.4^C^
+ 1-MCP after storage	77.9^C^
**+ Preharvest 1-MCP**	
No 1-MCP	80.6^B^
1-MCP at harvest	81.5^AB^
+ 1-MCP after storage	82.3^A^
*Significance* *^1^*	****

Values are averages from both storage regimes plus 1, 7, and 14 days at room temperature (21–22°C).

1 **** = significant at *p* < 0.0001.Values within a column with the same letter are not significantly different at *p* < 0.05.

During the second year of study, there was no overall significant effect of postharvest 1-MCP on firmness in ‘Gala’ apples, with averages ranging from 71.6 to 72.7 N. Moreover, there was no significant interaction of postharvest 1-MCP and storage regimes on fruit firmness after storage, plus during the 14 days at room temperature (data not presented). Firmness was overall higher in fruit held in 0.5 kPa O_2_ compared to similar fruit held in either 1.0 or 1.5 kPa O_2_ after 8 months of storage (Table 4).

**Table 4:** Overall quality of ‘Gala’ apples stored in 1.5, 1.0, or 0.5 kPa O_2_ + <0.5 kPa CO_2_ for at 0.5°C for 8 months in Year 2.

	Internal ethylene concentration (μl L^−1^)	Firmness (*N*)	Malic acid^1^ (mg)	Greasiness^2^ (1–3)
1.5 kPa O_2_	0.37^A^	71.3^B^	348.75^C^	1.22^A^
1.0 kPa O_2_	0.39^A^	70.7^B^	364.80^B^	1.17^B^
0.5 kPa O_2_	0.23^B^	74.2^A^	401.31^A^	1.11^C^
*Significance* *^3^*	****	***	****	****

Values are averages from after storage plus 1, 7, and 14 days at room temperature (21–22°C).

1 mg per 100 ml of juice.

2 Greasiness scale: 1 = none to slight greasiness; 2 = moderate greasiness (noticeable by feel); and 3 = severe greasiness (slippery, difficult to hold).

3 ***, **** = significant at *p* < 0.001 or *p* < 0.0001, respectively. Values within a column with the same letter are not significantly different at *p* < 0.05.

Internal ethylene concentrations were less than 0.2 μl L^−1^ in apples from all treatment combinations after 9 months of storage plus 1 and 7 days at room temperature in the first year. After 14 days at room temperature, apples treated with postharvest 1-MCP at harvest had consistently lower internal ethylene than those not treated with postharvest 1-MCP (Table 5), regardless of preharvest 1-MCP and storage regimes. Apples held in 1.5 kPa O_2_ and treated with postharvest 1-MCP after storage also had lower internal ethylene concentration than those not treated with postharvest 1-MCP, whereas this effect was not found in fruit held in 0.6 kPa O_2_. There was no significant effect of preharvest 1-MCP on internal ethylene concentrations.

**Table 5:** Internal ethylene concentration of ‘Gala’ apples treated without or with preharvest 1-MCP orchard spray at 7 days before harvest in combination without or with postharvest 1-MCP (1 μl L^-1^) for 24 h at harvest or after storage in 1.5 or 0.6 kPa O_2_ + <0.5 kPa CO_2_ at 0.5°C for 9 months in Year 1.

	Internal ethylene concentration (μl L^−1^)
***1.5 kPa O_2_***	
**No preharvest 1-MCP**	
No 1-MCP	9.534^A^
+ 1-MCP at harvest	1.526^B^
+ 1-MCP after storage	1.168^B^
**+ Preharvest 1-MCP**	
No 1-MCP	10.375^A^
+ 1-MCP at harvest	1.901^B^
+ 1-MCP after storage	1.112^B^
***0.6 kPa O_2_***	
**No preharvest 1-MCP**	
No 1-MCP	7.908^A^
+ 1-MCP at harvest	0.467^B^
+ 1-MCP after storage	10.191^A^
**+ Preharvest 1-MCP**	
No 1-MCP	7.686^A^
+ 1-MCP at harvest	0.557^B^
+ 1-MCP after storage	4.997^AB^
*Significance* *^1^*	****

Values are averages from after storage plus 1, 7, and 14 days at room temperature (21–22°C).

1**** = significant at *p* < 0.0001.Values within a column with the same letter are not significantly different at *p* < 0.05.

Internal ethylene concentration was only affected by O_2_ concentration in the second year, regardless of postharvest 1-MCP treatment; apples held in 0.5 kPa O_2_ had less internal ethylene than those held in either 1.0 and 1.5 kPa O_2_ (Table 4). Internal ethylene concentrations were less than 0.1 μl L^−1^ in apples from all treatments after storage plus 1 and 7 days at room temperature, and remained less than 1 μl L^−1^ after 14 days at room temperature in all apples.

After storage, soluble solids concentration and malic acid content were slightly higher in ‘Gala’ apples with preharvest 1-MCP compared to similar fruit not sprayed with preharvest 1-MCP; overall soluble solids of 12.1 vs. 11.9% and malic acid content of 589 vs. 574 mg per 100 ml of juice, respectively. There were little and inconsistent effects of postharvest 1-MCP on soluble solids concentration and malic acid content, and there were no interactions with low O_2_ level (data not presented). In the second year, malic acid content decreased and greasiness increased with higher O_2_ concentrations (Table 4).

There was no greasiness in apples treated with postharvest 1-MCP at harvest time and then held in 0.5 kPa O_2_ for 8 months plus 14 days at room temperature; this was significantly less than those treated with postharvest 1-MCP after storage or without 1-MCP treatment (Table 6). Greasiness was more severe after storage plus 14 days at room temperature in apples stored in higher O_2_ concentrations of 1.0 or 1.5 kPa (Tables 4, 6). At these higher O_2_ concentrations, postharvest 1-MCP consistently reduced greasiness, regardless of application timing and storage regime. There was no greasiness found in apples during the first year of this study.

**Table 6:** Greasiness of ‘Gala’ apples treated without or with postharvest 1-MCP (1 μl L^-1^) for 24 h at harvest or after storage in 1.5, 1.0 or 0.5 kPa O_2_ + <0.5 kPa CO_2_ at 0.5°C for 8 months plus 14 days at room temperature (21–22°C) in Year 2.

	Greasiness^1^ (1–3)
**1.5 kPa O_2_**	
No 1-MCP	2.00^A^
+ 1-MCP at harvest	1.50^C^
+ 1-MCP after storage	1.50^C^
**1.0 kPa O_2_**	
No 1-MCP	1.80^B^
+ 1-MCP at harvest	1.37^D^
+ 1-MCP after storage	1.28^E^
**0.5 kPa O_2_**	
No 1-MCP	1.57^C^
+ 1-MCP at harvest	1.00^F^
+ 1-MCP after storage	1.50^C^
*Significance* *^2^*	****

1 Greasiness scale: 1 = none to slight greasiness; 2 = moderate greasiness (noticeable by feel); and 3 = severe greasiness (slippery, difficult to hold).

2 **** = significant at *p* < 0.0001.

Values within a column with the same letter are not significantly different at P < 0.05.

Incidence of stem-end cracking in ‘Gala’ apples was 8.6 and 3.9% overall in the first and second year of study, respectively. There were no significant effects due to preharvest or postharvest 1-MCP treatments or low O_2_ storage regimes on stem-end cracking (data not presented).

## Discussion

Reducing O_2_ levels to limit ethylene-induced fruit ripening processes and maintain fruit quality, while mitigating risks associated with low O_2_-related stress in apples, is a challenge for long-term storage. Internal browning was reduced in ‘Gala’ apples with lower O_2_ levels during storage in both years of this study. Thewes et al. (2021) also found that lowering O_2_ conditions reduced flesh breakdown in ‘Royal Gala’ apples, along with limiting ACC oxidase activity and internal ethylene concentration, when held in 1.2–0.4 kPa O_2_ at 1°C for 9 months plus 1 week shelf-life. In contrast, there was no difference in incidence of flesh breakdown in ‘Braeburn’ apples from 1.2 kPa O_2_ and DCA storage regimes with less than 0.5 kPa O_2_ (Schmidt et al., 2020).

Internal browning, reported as diffuse flesh breakdown or radial stem-end flesh breakdown in ‘Royal Gala’ apples, can be influenced by postharvest 1-MCP treatment and temperature in air storage at 0.5 or 3°C for up to 6 months (Lee et al., 2016). 1-MCP at harvest time reduced diffuse flesh breakdown but enhanced sensitivity to radial stem-end flesh breakdown. Ethylene inhibition affected internal browning development in ‘Gala’ apples in this study, but the effect varied depending on the timing of 1-MCP treatment and low O_2_ level during storage. Overall, there was little effect of 1-MCP on internal browning in 0.5–0.6 kPa O_2_, even though there was high incidence in the second year of study.

Treatment with 1-MCP either preharvest or postharvest has been shown to affect some physiological disorders in apples differently. Preharvest 1-MCP reduces soft scald in ‘Honeycrisp’ apples, whereas postharvest 1-MCP has little effect on disorders in ‘Honeycrisp’ (DeEll and Ehsani-Moghaddam, 2010; DeEll et al., 2016a; Shoffe et al., 2021). Preharvest 1-MCP increases susceptibility of ‘Honeycrisp’ apples to the onset of bitter pit and decreases susceptibility to senescent breakdown, while the combination of preharvest and postharvest 1-MCP increases leather blotch (Shoffe et al., 2021). Both pre- and postharvest 1-MCP treatments exacerbate external CO_2_ injury in ‘Empire’ apples (DeEll and Ehsani-Moghaddam, 2012b).

Treatment with preharvest and postharvest 1-MCP after storage in 1.5 kPa O_2_ reduced internal browning, compared to apples with only preharvest 1-MCP; however, this effect was not found when 1-MCP was applied at harvest instead of after storage. Previous studies have shown postharvest 1-MCP at harvest time alone can exacerbate the onset of flesh browning in ‘Empire’ apples (Fawbush et al., 2008; Jung and Watkins, 2011). Furthermore, ‘Empire’ apples treated with 1-MCP at harvest developed a higher incidence of flesh browning compared to fruit without 1-MCP after 8 months of CA storage in 1.5 and 2.5 kPa O_2_, whereas the use of RQ-based DCA storage with 0.6 kPa O_2_ limited flesh browning to less than 1% incidence (DeEll and Lum, 2017). Results from the current study suggest effective 1-MCP treatment to reduce internal browning in ‘Gala’ apples involves two applications of 1-MCP, where one is applied preharvest and another after storage. However, the absence of ethylene action and its underlying physiological role for internal browning development remains to be elucidated.

There was notably more internal browning in ‘Gala’ apples from the second year, with lower incidence in apples from lower O_2_ regimes. The onset of internal browning or flesh browning in apples can be influenced by fruit maturity at harvest, where apples with advanced maturity or harvested at later dates tend to have higher incidence (DeEll et al., 2008; Thewes et al., 2017). Incidence of internal browning also varies among years and orchards (Watkins and Liu, 2010; Jung and Watkins, 2011; DeEll and Ehsani-Moghaddam, 2012a; Deyman et al., 2014; DeEll and Lum, 2017). ‘Gala’ apples in the second year of this study had advanced maturity at harvest, as indicated by high starch values and soluble solids concentration. This would have likely contributed to the high incidence of internal browning in second year.

The low incidence of stem-end browning in ‘Gala’ apples during both years of this study could be due to the long storage duration, in which stem-end browning had time to radiate further into the flesh. However, Lee et al. (2016) suggested internal browning (diffuse flesh breakdown) and stem-end browning (radial stem-end flesh breakdown) are two separate disorders. Preliminary results, with ‘Gala’ apples from the same orchard as used in this study, showed no internal browning or stem-end browning after 4–5 months of storage in DCA-RQ (low of 0.4 kPa O_2_, using SafePod technology) at 0.5°C, while there was 17 and 6% incidence of stem-end browning in 2.5 and 1.5 kPa O_2_, respectively (DeEll, unpublished data). Nock et al. (2019) also found less stem-end browning in ‘Gala’ (Fulford strain) with lower O_2_ storage (0.5 vs. 2 kPa).

The beneficial effect of 1-MCP on firmness retention in apples has been well documented (Watkins et al., 2000; Jung and Watkins, 2011; DeEll et al., 2007, 2016a; DeEll and Lum, 2017). There was no overall significant effect of postharvest 1-MCP treatment (at harvest or after storage) on fruit firmness in the current study and there was no interaction with low O_2_ levels. However, preharvest 1-MCP maintained greater firmness retention in ‘Gala’ apples compared to fruit without preharvest 1-MCP. Application of 1-MCP in the orchard prior to harvest has also been shown to enhance firmness retention after standard CA storage in ‘Scarletspur Delicious’ and ‘Cameo’ apples (Elfving et al., 2007).

There was improvement in firmness retention when preharvest 1-MCP was applied, but no such effect was found with postharvest 1-MCP treatment. Preharvest 1-MCP delayed starch hydrolysis prior to harvest (Table 1), which can lead to enhanced firmness retention during and after removal from storage (Elfving et al., 2007; Yuan and Carbaugh, 2007; DeEll and Ehsani-Moghaddam, 2010; Yingjie et al., 2021). Preharvest 1-MCP can also have inconsistent effects on starch hydrolysis or firmness retention at harvest (Yingjie et al., 2021) or during and after removal from storage depending on the apple cultivar, growing season, concentration, and timing of preharvest treatment (McArtney et al., 2009; Doerflinger et al., 2019).

Increased respiration rate and ethylene production can associate with loss in fruit firmness retention (Both et al., 2017). ‘Gala’ apples with preharvest 1-MCP treatment appeared to have reduced respiration rate immediately after harvest, compared to fruit without preharvest 1-MCP (Figure 1). This may in part explain the enhanced firmness retention demonstrated by preharvest 1-MCP. However, the respiration measurements of fruit with and without preharvest 1-MCP were from a single replicate and one growing season. This aspect warrants further investigation.

Previous studies have demonstrated that lowering O_2_ levels during storage can enhance firmness retention in apple (Veltman et al., 2003; Köpcke, 2015; Rebeaud and Gasser, 2015; DeEll and Lum, 2017). Enhanced firmness retention due to lowering O_2_ levels during storage and without 1-MCP treatment was also found in ‘Gala’ apples in this study. Lowering O_2_ levels during storage can reduce activity of the ethylene biosynthesis enzyme, 1-aminocyclopropane-1-carboxylate (ACC) oxidase (Thewes et al., 2015, 2017, 2021). Fruit firmness retention can be enhanced due to reduced availability of ethylene, which can impact cell wall modification and degradation genes and enzymes that are ethylene-regulated (Bennett and Labavitch, 2008; Gwanpua et al., 2016; Win et al., 2021).

As expected, lower O_2_ levels alone during storage reduced ethylene production in ‘Gala’ apples without the application of 1-MCP in both years of this study. This is consistent with previous studies where ethylene production in apples decreases with lower O_2_ levels in storage (Lumpkin et al., 2014; Thewes et al., 2015, 2017, 2021; DeEll and Lum, 2017). Moreover, postharvest 1-MCP treatment at harvest consistently reduced internal ethylene concentration in ‘Gala’ apples compared to untreated fruit, which aligns with previous study by Lee et al. (2016). Ethylene inhibition interacted with low O_2_ concentration in the current study, as ‘Gala’ apples held in 1.5 kPa O_2_ and treated with 1-MCP after storage had less internal ethylene than fruit without postharvest 1-MCP, whereas this effect was not found in similar fruit held in 0.6 kPa O_2_. This lack of effect with 1-MCP after storage on internal ethylene concentration was also observed in ‘Empire’ apples held in 0.6 kPa O_2_ and treated with 1-MCP after storage (DeEll and Lum, 2017). However, ‘Empire’ treated with 1-MCP at harvest or after storage and held in 1.5 kPa O_2_ had reduced ethylene production compared to similar fruit with no 1-MCP. Treatment with 1-MCP after storage in 0.5–0.6 kPa O_2_ may not be effective due to lack of binding sites or ethylene presence. Efficacy of 1-MCP treatment during or after storage seems to be influenced by low O_2_ conditions, but the underlying biochemical processes remains to be further explored and confirmed.

The influence of postharvest 1-MCP for inhibiting ethylene production in ‘Gala’ apples was apparent in the first year but not in the second year of this study. Ethylene in ‘Gala’ from the first year was notably higher after removal from storage, compared to fruit from the second year. The effect of 1-MCP on inhibiting ethylene production could vary depending on whether apples harvested within a growing season have inherent elevated or low internal ethylene, where 1-MCP inhibition of ethylene production tends to be stronger in years with higher ethylene producing apples.

Postharvest 1-MCP treatment is known to have inconsistent or little effect on soluble solids concentration in apples (Watkins et al., 2000; DeEll and Ehsani-Moghaddam, 2012a; DeEll and Lum, 2017). Similarly, preharvest 1-MCP had no significant effect on soluble solids in ‘Honeycrisp’ apples (DeEll and Ehsani-Moghaddam, 2010). ‘Gala’ apples with preharvest 1-MCP had slightly higher soluble solids and malic acid after storage in the present study, but there was no interaction with low O_2_ levels. This suggests preharvest 1-MCP treatment alone can influence sugar and organic acid metabolism in long-term storage. Previous study has shown marginal higher malic acid content in postharvest 1-MCP-treated apples held in 1.5 or 2.5 kPa O_2_ plus 14 days at room temperature, compared to those with no 1-MCP (DeEll and Lum, 2017).

Timing of ethylene inhibition interacted with low O_2_ level to affect greasiness in ‘Gala’ apples during long-term storage. 1-MCP treatments have been shown to reduce greasiness in apples (Fan et al., 1999; Nock and Watkins, 2013; Lee et al., 2019; Shoffe et al., 2021), whereas results from the current study found 1-MCP treatment at harvest time and long-term storage in 0.5–0.6 kPa O_2_ eliminated greasiness development, plus for up to 14 days thereafter at room temperature. Peel greasiness can become more prevalent in apples with advanced maturity due to later harvested fruit and/or held in storage for prolonged duration (Watkins et al., 2005; DeLong et al., 2006). In the present study, ‘Gala’ apples harvested in the second year had more advanced maturity at harvest, as indicated by high starch values and soluble solids concentration. This may have led in part to more greasiness in the second year.

In summary, storage regimes with less than 1 kPa O_2_ provided the least amount of internal browning and best quality attributes. Ethylene inhibition provided further benefits, but this was dependent on the timing of 1-MCP treatment.

## Data Availability Statement

The original contributions presented in the study are included in the article/supplementary material; further inquiries can be directed to the corresponding author.

## Author Contributions

JD conceptualized and supervised the project and managed 1-MCP treatments and storage regimes. SL and YM conducted fruit evaluations and data compilation. GL and YM performed the data analyses. JD and GL wrote and prepared the manuscript. All authors contributed to the article and approved the submitted version.

## Funding

This project was funded in part through the Canadian Agri-Science Cluster for Horticulture 3, in cooperation with Agriculture and Agri-Food Canada’s AgriScience Program, a Canadian Agricultural Partnership initiative, the Canadian Horticultural Council and industry contributors.

## Conflict of Interest

The authors declare that the research was conducted in the absence of any commercial or financial relationships that could be construed as a potential conflict of interest.

## Publisher’s Note

All claims expressed in this article are solely those of the authors and do not necessarily represent those of their affiliated organizations, or those of the publisher, the editors and the reviewers. Any product that may be evaluated in this article, or claim that may be made by its manufacturer, is not guaranteed or endorsed by the publisher.
